# Human Cytomegalovirus and Human Herpesvirus 6 Coinfection of Dermal Fibroblasts Enhances the Pro-Inflammatory Pathway Predisposing to Fibrosis: The Possible Impact on Systemic Sclerosis

**DOI:** 10.3390/microorganisms10081600

**Published:** 2022-08-08

**Authors:** Irene Soffritti, Maria D’Accolti, Clara Maccari, Francesca Bini, Eleonora Mazziga, Flora de Conto, Adriana Calderaro, Maria-Cristina Arcangeletti, Elisabetta Caselli

**Affiliations:** 1 Section of Microbiology, Department of Chemical, Pharmaceutical and Agricultural Sciences, and LTTA, University of Ferrara, 44121 Ferrara, Italy; 2 Department of Medicine and Surgery, University of Parma, 43126 Parma, Italy

**Keywords:** systemic sclerosis, tissue fibrosis factors, HCMV, HHV-6

## Abstract

Systemic sclerosis (SSc) is a severe autoimmune disease likely triggered by genetic and environmental factors, including viral infections. Human cytomegalovirus (HCMV) and human herpesvirus 6A species (HHV-6A) have been associated with SSc, based on in vivo and in vitro evidence, but the data are still inconclusive. Furthermore, despite both viruses being highly prevalent in humans and able to exacerbate each other’s effects, no data are available on their joint effects. Hence, we aimed to study their simultaneous impact on the expression of cell factors correlated with fibrosis and apoptosis in in vitro coinfected fibroblasts, representing the main target cell type in SSc. The results, obtained by a microarray detecting 84 fibrosis/apoptosis-associated factors, indicated that coinfected cells underwent higher and more sustained expression of fibrosis-associated parameters compared with single-infected cells. Thus, the data, for the first time, suggest that HCMV and HHV-6A may cooperate in inducing alterations potentially leading to cell fibrosis, thus further supporting their joint role in SSc. However, further work is required to definitively answer whether β-herpesviruses are causally linked to the disease and to enable the possible use of targeted antiviral treatments to improve clinical outcomes.

## 1. Introduction

Systemic sclerosis (SSc), also known as scleroderma, is a rare autoimmune disease characterized by the hardening and tightening of the skin, with the concurrent involvement of internal organs, blood vessels, and the digestive tract. Included among connective tissue diseases, SSc is often categorized as “limited” or “diffuse”, which refers to the degree of skin involvement, as both types can involve vessels or organs, whereas localized SSc usually preferentially affects the skin [1]. Among internal organs, SSc can affect the heart or lungs, causing scarring due to fibrosis of the parenchymal cells, as well as an increase in blood pressure due to vessels’ stiffening.

SSc is generally characterized by immune alterations, micro-angiopathy, and massive production of extracellular matrix by altered fibroblasts, which represent the main target cells of the disease. Although SSc signs and symptoms vary from person to person, nearly every patient experiences hardening and tightening of the skin, with the fingers, hands, feet, and face the first parts of the body to be affected [2,3]. Raynaud’s phenomenon is a common symptom in SSc, related to the inappropriate contraction of the small blood vessels of the skin. Alongside tissue fibrosis, apoptosis also appears to be a key mechanism involved in all stages of the disease [4]. Fibrosis and apoptosis show several links in SSc, as increased serum levels of the soluble form of the proapoptotic protein Fas have been reported in SSc patients [5]; pathological fibroblasts show resistance to Fas apoptosis [6], and mice lacking Fas or Fas ligand (FasL) genes [7], or treated with anti-FasL antibodies [8], show diminished apoptosis and a concomitant decrease in fibrosis and collagen accumulation. Sera from SSc patients can induce apoptosis by stimulating Fas [9] or activating caspase-3, another important apoptosis mediator [10]. IL6, which is strongly increased in SSc, appears to be one of the major links between apoptosis and fibrosis, increasing TGFβ signaling and collagen synthesis [11], and by inducing apoptosis resistance in pathological fibroblasts and increased sensitivity in normal fibroblasts [12].

The causative factors of the disease have not yet been elucidated, though SSc is hypothesized to develop due to both genetic predisposition and environmental agents, including viral infections [3]. Among viruses whose infection has been potentially associated with SSc, herpesviruses have been studied for several decades, with research showing that the beta-herpesviruses human cytomegalovirus (HCMV) and human herpesvirus 6 (HHV-6) seem particularly involved in the onset/progression of the disease [13,14,15,16,17]. Both viruses are highly prevalent in the human population, causing primary infection during early childhood [18,19]. Following resolution of primary infection they establish a long-life latent infection in the host, with asymptomatic reactivations in the immunocompetent adult but potentially severe symptomatic diseases in the immunocompromised host, among whom both viruses have been associated with several autoimmune diseases, including connective tissue diseases [16,17,20,21,22,23,24,25]. 

In fact, HCMV and HHV-6 transcripts have been detected in the skin of SSc patients [15,26], and significantly higher levels of anti-HCMV-UL94 and anti-HHV-6-U94 antibodies were observed in SSc subjects, compared with controls [15,27,28,29,30]. HHV-6 is associated with Hashimoto’s thyroiditis, a disease often preceding the development of SSc [31], and the presence of specific anti-HCMV CD8+ T cells is associated with SSc disease severity [14]. Of note, SSc autoantibodies bind HCMV UL94 and enhance the apoptosis of endothelial cells [9,27].

Furthermore, both viruses have a tropism for endothelial and fibroblast cells, and both are deeply involved in SSc disease [3,26,32,33,34,35]. In vitro infection with HCMV and HHV-6 induces the expression of pro-fibrotic factors in primary human dermal fibroblasts [13] and modulates the expression of miRNAs, recognized for their key function in fibrosis [36].

Since these two viruses are ubiquitously present in the human population, they are often simultaneously reactivated in the adult host: they can exacerbate each other, especially in immunosuppressed subjects [37,38,39], and their co-reactivation can finally lead to a worse clinical outcome in critically ill patients [39,40]. 

Based on these observations, we wanted to assess whether coinfection by HCMV and HHV-6 could trigger significantly higher levels of factors potentially leading to tissue fibrosis or cell apoptosis, than either virus alone. To this end, primary human dermal fibroblasts were individually infected or coinfected with HCMV and HHV-6, and the expression of cell factors correlated to fibrosis and apoptosis was analyzed.

## 2. Materials and Methods

### 2.1. Tissue Cultures and Virus Strains

Primary human dermal fibroblast cultures (NHDF-Ad, CC-2511; Lonza, Basel, Switzerland) were propagated as previously described [36,41] in a complete fibroblast cell medium (fibroblast cell basal medium, FCBM), with the addition of 2% fetal bovine serum (FBS), 0.1% r-human fibroblast growth factor-B, 0.1% insulin, and 0.1% gentamicin sulphate/amphotericin-B (Clonetics™ FGM™-2 Bullet Kit™, Lonza, Basel, Switzerland). At around 80% confluence, the cells were detached and seeded using a ReagentPack Subculture Reagent Kit (Lonza, Basel, Switzerland), according to the manufacturer’s instructions. MRC5 fibroblast cells (ECACC 05072101, Merck Life Science, Milan, Italy) were grown in Earle’s modified Minimum Essential Medium (EMEM) supplemented with 1% l-glutamine, 1% non-essential amino acids, and 10% FBS. Human J-Jhan T cells were grown in suspension in an RPMI-1640 medium (Lonza, Basel, Switzerland) supplemented with 10% FBS, as already described [42].

The TB40E HCMV strain was kindly provided by Professor Thomas Mertens (Institute of Virology, Ulm University, Germany). The virus was propagated in MRC5 fibroblasts and the virus titer (10^9^ PFU/mL) was determined as previously described [36]. The U1102 strain of HHV-6A was grown in J-Jhan T cells as previously described [42] and titrated by real time quantitative polymerase chain reaction (qPCR), as previously described [42]. The virus titer of the stock used in all the infections corresponded to 10^10^ genome equivalents/mL.

### 2.2. Viral Infection

Primary human dermal fibroblasts were grown until 90% confluence, and infected with HCMV TB40E at a multiplicity of infection (MOI) of 0.1 PFU/cell, as previously described [36]. A MOI of 1.0 genome equivalent/cell was used in the infections with U1102 HHV-6A, as already described [13,36]. After 2 h of adsorption at 37 °C, virus inocula were removed and replaced with complete FCBM. Cells were then incubated at 37 °C and collected by scraping 0, 1, 2, 4, 7, and 10 days post infection (d.p.i.). Cell samples were then centrifuged at 1000× *g* for 5 min at 4 °C to collect cell pellets, which were washed in PBS to eliminate eventual secreted factors, frozen in liquid nitrogen, and kept at −80 °C until use. Duplicate samples were tested for each series.

### 2.3. Nucleic Acid Extraction

Extraction of total nucleic acids from 10^6^ infected cells was performed with an AllPrep DNA/RNA/miRNA kit (Qiagen, Hilden, Germany), allowing us to simultaneously obtain the total DNA and RNA from any individual sample. 

### 2.4. DNA Analysis: Virus Quantitation in Infected Cells

Total DNA was used to assess and quantify both viruses’ genomic DNA inside the infected cells. Briefly, HCMV’s DNA presence was analyzed by a qPCR CMV ELITe MGB^®^ Kit (ELITechGroup, Turin, Italy), targeting the HCMV DNA exon 4 region of the immediate–early (IE)1 gene, in a 7500 Real-Time PCR system (ABI PRISM, Applied Biosystems, Milan, Italy), and following the manufacturer’s instructions, as previously described [13]. HHV-6A was quantified by a specific qPCR designed to amplify the U94 gene, using a Quant Studio 5 thermocycler (Thermo Fisher Scientific, Milan, Italy), as already described [31]. Amplification of the house-keeping human RNase P gene was performed as a control and used to normalize virus counts to cell number. The results were expressed as the DNA genome copy number per µg of total DNA extracted from cells.

### 2.5. RNA Analysis

Extracted RNA was first treated to eliminate any eventual contaminant DNA by DNase I digestion (Thermo Fisher Scientific, Waltham, MA, USA). The absence of DNA was checked by PCR amplification of the human β-actin gene using 10 ng of extracted RNA as a template. 

For the expression of fibrosis or apoptosis cell factors, extracted RNA was reverse transcribed by the RT2 First Strand kit and analyzed by a qPCR microarray simultaneously detecting 84 factors associated with fibrosis or apoptosis (both from Qiagen, Hilden, Germany), following the manufacturer’s protocol [15]. Microarray plates were run on a Quant Studio 5 real-time PCR system (Thermo Fisher Scientific, Milan, Italy), in accordance with the manufacturer’s instructions. Results were analyzed by Qiagen Gene Globe software (https://geneglobe.qiagen.com/ca/analyze; accessed on 20 May 2022) and expressed as a fold-change compared with the values detected in control uninfected cells, after normalizing toward the six included housekeeping factors (beta-actin, beta2-microglobulin, GADPH, HPRT1, RPLP0, and HGDG). Duplicate samples from two independent experiments were analyzed and the analysis threshold was at 2-fold change of up- or down-regulation.

### 2.6. Statistical Analyses

Statistical analyses were performed with Graph Pad Prism 9 software. The Mann–Whitney test, Fisher’s exact test, and paired t-test were used to determine the significance of differential factor expression between infected and control cells. Bonferroni correction for multiple comparisons was applied and a *p* value < 0.05 was considered statistically significant.

## 3. Results

### 3.1. HCMV and HHV-6A Coinfection of Primary Human Dermal Fibroblasts

Based on previous results indicating that HHV-6A was detectable in the skin of SSc patients [15], thus supporting its higher tissue tropism compared with the -6B species, the infection of primary dermal fibroblasts was performed using the HHV-6A species, in the presence or absence of HCMV. HCMV and HHV-6A were used at 0.1 and 1 MOI, respectively, since these amounts are most closely related to natural infections in vivo. Uninfected, single-infected and double-infected cells were then collected at 0 (immediately after adsorption), 1, 2, 4, 7, and 10 d.p.i. to assess the amount of intracellular virus by qPCRs specifically targeting the HCMV *IE-1* gene and the HHV-6 *U94* gene.

The results, as summarized in Table 1, confirmed that primary human dermal fibroblasts are permissive for HCMV and HHV-6A replication, as already shown by previous studies. Interestingly, coinfection enhanced the replication of both viruses, as judged by the amount of viral DNA detected in coinfected versus single-infected cells. In fact, viral genome copies increased by about 1 Log compared with HCMV and HHV-6A single infection, thus supporting their ability to enhance each other when simultaneously present in infected cells. Different from HCMV, however, which continued to lytically infect cells until 10 d.p.i., HHV-6A likely established a latent infection in fibroblasts after 7 d.p.i, as suggested by its stably decreasing number of genome copies per µg of total DNA extracted from infected cells. 

**Figure 1:**
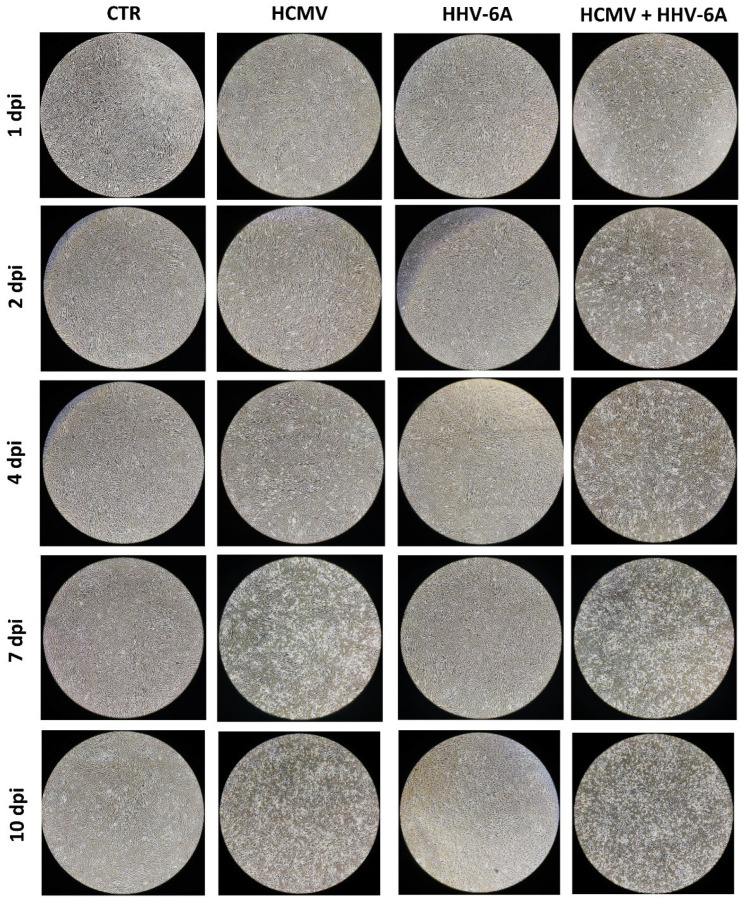
Primary human dermal fibroblasts infected with HCMV and HHV-6A. Images were acquired with a phase-contrast microscope at 4X original magnification, and are representative of sample duplicates in two independent experiments.

Cytopathic effect (CPE) was only observed in HCMV infected cells (both single and double-infected), whereas no CPE was detected in fibroblasts infected with HHV-6A alone. Interestingly, the simultaneous presence of both β-herpesviruses caused an earlier appearance of the HCMV-induced CPE, as shown in Figure 1. In fact, while HCMV alone caused evident CPE starting from 4 d.p.i., the concomitant presence of HHV-6A, despite the absence of CPE induced by this virus, induced a detectable CPE at 2 d.p.i., suggesting the potential of inducing worse effects in infected cells when the two viruses are simultaneously present.

### 3.2. Effect of HCMV and HHV-6A Coinfection on the Expression of Fibrosis-Associated Factors

**Figure 2:**
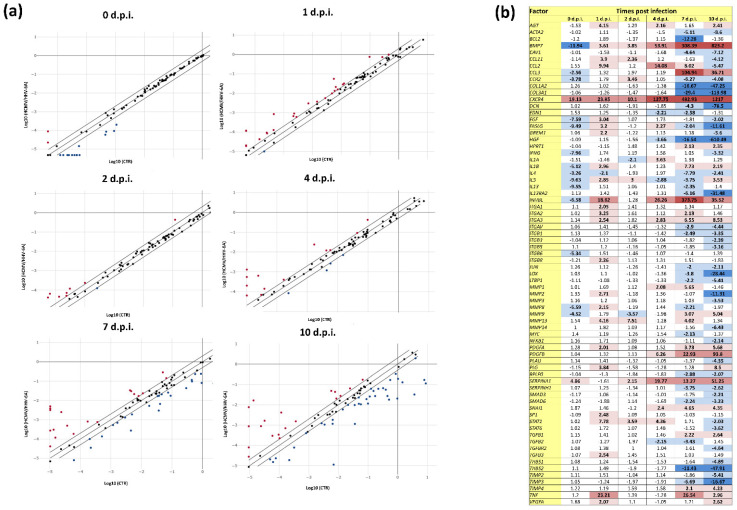
Expression of fibrosis-associated factors in response to HCMV/HHV-6A coinfection of human dermal fibroblasts. Cell samples were collected at the indicated days post infection (d.p.i.) and analyzed by qPCR microarray. (**a**) Scatterplot representation: the threshold was 2-fold change in infected vs. control cells; red and blue dots represent up-regulated and down-regulated factors, respectively; results are expressed as mean values of duplicate samples in two independent experiments. (**b**) Detailed values of down- and up-regulated factors: dark blue, down-regulation >10-fold; light blue, down-regulation between 2- and 9.9-fold; light red, up-regulation between 2- and 9.9-fold; red, up-regulation between 10- and 99.9-fold; dark red, up-regulation >100-fold. All values above the 2-fold threshold are indicated in bold.

The analysis of the expression pattern of fibrosis-associated factors, as judged by qPCR microarray analysis on cell extracted RNA, showed the presence of profound alterations in infected cells compared with uninfected controls (Figure 2), as HCMV/HHV-6A coinfection induced the modulation of several factors at all tested time points. In particular, among the 84 factors included in the microarray analysis, the expression of 16 factors were altered just after viruses’ adsorption, and 26, 10, 17, 46 and 57 factors were activated or repressed at 1, 2, 4, 7, and 10 d.p.i., respectively (Figure 2).

Some factors were highly induced at most times points, while others showed different temporal regulation. Among the factors that appeared to be constantly up-regulated, focusing on those that increased at least 10-fold compared with controls, were bone morphogenic protein 7 (BMP7, up to 823.2-fold), chemokine receptor type 4 (CXCR4, up to 1217-fold), inhibin β subunit-E (INHBE, up to 373.75-fold), and SERPINA 1 (up to 51.25-fold). Other significantly up-regulated factors (p_c_ < 0.05) at one or more time points included chemokine (C-C motif) ligand-2 (CCL2, induced up to 14.08-fold at 1, 4, and 7 d.p.i.), chemokine (C-C motif) ligand-3 (CCL3, induced up to 104.94-fold at 7 and 10 d.p.i.), platelet-derived growth factor subunits A and B (PDGFA and PDGFB, induced up to 93.8-fold between 4 and 10 d.p.i.), and the proinflammatory cytokine tumor necrosis factor-α (TNFα, induced up to 26.54-fold at 1, 7, and 10 d.p.i.). 

Down-regulated factors appeared to be concentrated at later time points (7 and 10 d.p.i.) (Figure 2b). The most inhibited factors (a >-10-fold down-regulation compared with controls) included COL1A2 (-7.25-fold), COL3A1 (-113.98-fold), DCN (-78.5-fold), FASLG (-11.61-fold), HGF (-610.49-fold), IL13RA2 (-31.48-fold), LOX (-28.44-fold), MMP2 (-11.31-fold), THBS2 (-47.91-fold), and TIMP3 (-16.67-fold). 

The results obtained in fibroblasts individually infected with HCMV and HHV-6A (Table S1) confirmed those obtained in previous studies, showing that HCMV could up-regulate BMP7 (from 4 to 10 d.p.i.), CCL2 (at 4 and 7 d.p.i.), CCL3 (at 2, 4, and 10 d.p.i.), CXCR4 (from 1 to 10 d.p.i.), SERPINA1 (from 4 to 10 d.p.i.), IL1β (at all time points), IL13 (at 10 d.p.i.), IL-13 receptor subunit alpha 2 (IL13RA2, from 4 to 10 d.p.i.), MMPs and TNFα (from 4 to 10 d.p.i.). HHV-6A infection was, instead, essentially altering the expression of BMP7 (from 4 to 10 d.p.i.), CXCR4 (from 4 to 10 d.p.i.), IL1β (from 4 to 10 d.p.i.), IL4 (at 7 and 10 d.p.i.), IL10 (at 10 d.p.i.), SERPINA1 (at 7 and 10 d.p.i.), and TNFα (at 1, 7, and 10 d.p.i.). Notably, the expression pattern of some factors was significantly different in dual-infected cells compared with single-infected cells (Table S1). In fact, in HCMV/HHV-6A coinfected cells, BMP7 was constantly up-regulated at all time points, reaching a peak of expression at 10 d.p.i. (823.2-fold), whereas it was up-regulated constantly but to a lower extent in HHV-6A infected cells (up to 13.6-fold) and expressed at quite high levels at 10 d.p.i. in HCMV-infected cells (134.1-fold). Similarly, CXCR4 up-regulation was evident after virus adsorption in the coinfection (19.13-fold) and increased until the end of the experiment, reaching a 1217-fold peak of up-regulation at 10 d.p.i. compared with controls. Instead, the individually infected cells exhibited a lower CXCR4 induction, peaking at 4 d.p.i. in HHV-6A infection (21.04-fold) and at 10 d.p.i. in HCMV infection (123.08-fold). Even more interestingly, the INHBE factor, which was not induced in the single-infected cells, was instead highly expressed at all time points (except at 0 d.p.i.) as a result of double infection, peaking at 7 d.p.i. with a 373.75-fold up-regulation compared with control cells. Another differently induced factor was SERPINA1, which was overexpressed at all the time points in dual-infected fibroblasts (up to 51.25-fold at 10 d.p.i.), whereas it was poorly (10-fold at 4 d.p.i.) induced or not at all in HCMV- or HHV-6A-infected cells. 

### 3.3. Effect of HCMV and HHV-6A Coinfection on the Expression of Apoptosis-Associated Factors

**Figure 3:**
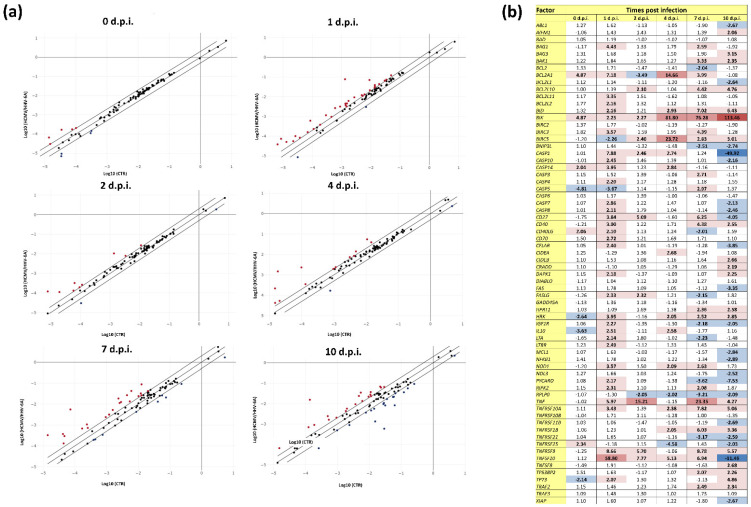
Expression of apoptosis-associated factors in response to HCMV/HHV-6A coinfection of human dermal fibroblasts. Cell samples were collected at the indicated days post-infection (d.p.i.) and analyzed by qPCR microarray. (**a**) Scatterplot representation: the threshold was at 2-fold change in infected vs. control cells; red and blue dots represent up-regulated and down-regulated factors, respectively; results are expressed as mean values of duplicate samples in two independent experiments. (**b**) Detailed values of down- and up-regulated factors: dark blue, down-regulation >10-fold; light blue, down-regulation between 2- and 9.9-fold; light red, up-regulation between 2- and 9.9-fold; red, up-regulation between 10- and 99.9-fold; dark red, up-regulation >100-fold. All values above the 2-fold threshold are indicated in bold.

Similar to what was observed for fibrosis factors, the expression of apoptosis-associated factors was also markedly altered by HCMV/HHV-6A coinfection in human dermal fibroblasts. Overall, immediately after adsorption, nine factors already appeared to be differently expressed compared with controls. Afterwards, with the beginning of virus replication, 35, 11, 15, 32, and 42 factors were up- or down-regulated, respectively, at 1, 2, 4, 7, and 10 d.p.i. (Figure 3).

In general, the alteration of expression of apoptosis-related factors was lower compared with that observed in fibrosis-associated factors. Factors highly upregulated (>10-fold) at all or most time points, included Bcl-2-interacting killer factor (BIK, up to 113.46-fold at 10 d.p.i.), and TNF (up to 23.35-fold at 7 d.p.i.). Other significantly up-regulated factors included BCL2-Like 10 ‘Apoptosis Facilitator’ (BCL2L10, up to 4.76 at 10 d.p.i.), BH3 interacting domain death agonist (BID, up to 7.02-fold at 7 d.p.i.), TNF-related apoptosis-inducing ligand receptor 1 (TNFRSF10A, up to 7.82-fold at 7 d.p.i.), TNFRSF1B (up to 6.03-fold at 7 d.p.i.), and TNFRSF9 (up to 8.78-fold at 7 d.p.i.). The CD27 and CD40 molecules, both belonging to the TNF-receptor superfamily, were also upregulated at various time points, with the highest up-regulation peaking at 7 d.p.i. (6.25- and 4.38-fold, respectively). The baculoviral IAP repeat containing 3 (BIRC3) was up-regulated at 1 and 7 d.p.i. (3.57- and 4.39-fold, respectively).

A biphasic trend was observed for some factors. The CD27 molecule, belonging to the TNF-receptor superfamily, was upregulated at 1, 2, and 7 d.p.i. (up to 6.25-fold at 7 d.p.i.) but down-regulated at 10 d.p.i. (-4.05-fold). TNFRSF10 was highly up-regulated at early time points (58.80-fold at 1 d.p.i.) but down-regulated at 10 d.p.i. (-11.46-fold). Two caspases (CASP1 and CASP14) were induced at 1 d.p.i. (7.88- and 3.95-fold) but were down-regulated or normally expressed at 10 d.p.i. (-49.92- and -1.11-fold). 

Altogether, factors that were solely down-regulated by HCMV/HHV-6A coinfection appeared to be altered only at low levels and at later time points (i.e., 10 d.p.i.); they included ABL proto-oncogene 1 (ABL1, -2.67-fold at 10 d.p.i.), B-cell CLL/lymphoma 2 (BCL2, -2.04-fold at 7 d.p.i.), Bcl-2-like protein 1 (BCL2L1, -2.64-fold at 10 d.p.i.), BCL2 interacting protein 3 like (BNIP3L, -2.51- and -2.74-fold at 7 and 10 d.p.i.), Fas cell surface death receptor (FAS, -3.35-fold at 10 d.p.i.), myeloid cell leukemia sequence 1 (MCL1, -2.84-fold at 10 d.p.i.), nuclear factor kappa B subunit 1 (NFKB1, -2.89-fold at 10 d.p.i.), nucleolar protein 3 (NOL3, -2.52-fold at 10 d.p.i.), ribosomal protein lateral stalk subunit P0 (RPLP0, from -2.05- to -3.21-fold at 2, 4, 7, and 10 d.p.i.), TNF receptor superfamily member 11b (TNFRSF11B, -2.69-fold at 10 d.p.i.), NF receptor superfamily member 21 (TNFRSF21, -3.19- and -2.59-fold at 7 and 10 d.p.i., respectively), and X-linked inhibitor of apoptosis (XIAP, -2.67-fold at 10 d.p.i.).

In individually infected cells, most apoptosis-related factors were up- or down-regulated at late time points, and some significant differences were observed between single and double-infected cells. The most evident differences included the constant up-regulation of BIK (which was only detected in coinfected cells), the up-regulation of BIRC3 (which instead showed a biphasic trend with early activation and late inhibition in single-infected cells), the earlier and constant up-regulation of TNFSF10 (which instead increased only at 1 and 2 d.p.i. in HCMV-infected cells, and was not significantly altered by HHV-6A alone), and the over-expression of CD27, which was observed until the end of experimentation in HCMV single-infected cells, whereas it appeared to be down-regulated in double-infected cells (Table S2).

## 4. Discussion

The β-herpesviruses HCMV and HHV-6 are ubiquitously present in humans worldwide, sharing common biological features with respect to the pathologies with which their infection has been associated in the adult host, often finally leading to worse clinical disease when co-reactivating in a susceptible subject [37,38,39,40]. Both viruses have been associated with the onset of SSc and other autoimmune diseases [14,15,26,27,28,29,30,31], and both exhibit a tropism for fibroblast cells [3,26,32,33,34,35], where their individual infection can induce the expression of pro-fibrotic factors and miRNAs [13,36]. Nevertheless, despite the fact that they are usually simultaneously present in the host, the two viruses have rarely been studied together to determine the effects of their coinfection.

The results arising from the present work show, for the first time, that the HCMV/HHV-6 in vitro coinfection of fibroblasts has a remarkable impact on the expression of factors associated with cell fibrosis and apoptosis. 

Namely, virus coinfection significantly up- or down-regulated at least 19 fibrosis-associated factors, including 9 up-regulated factors: BMP7 (+832.2-fold), CXCR4 (+1217-fold), INHBE (+373.75-fold), SERPINA1 (+51.25-fold), CCL2 (+14.08-fold), CCL3 (+104.94-fold), PDGFA and PDGFB (+93.8-fold), and TNFα (+26.54-fold); and 10 down-regulated factors: COL1A2 (-47.25-fold), COL3A1 (-113.98-fold), DCN (-78.5-fold), FASLG (-11.61-fold), HGF (-610.49-fold), IL13RA2 (-31.48-fold), LOX (-28.44-fold), MMP2 (-11.31-fold), THBS2 (-47.91-fold), and TIMP3 (-16.67-fold). Of note, almost all up-regulated factors had recognized pro-fibrotic action and were induced at a high level by coinfection rather than by individual viruses. CXCR4 was found to be overexpressed in the skin of SSc patients [43,44]. INHBE upregulation, which is associated with ER stress in human fibroblasts, can result from the overwhelming activation of TGF-β1 [45]. In fact, besides TGF-β itself, the TGF-β superfamily includes activins (including INHBE), growth and differentiation factors (GDFs), and bone morphogenetic proteins (BMPs), all possessing essential roles in early embryonic development and in regulating tissue homeostasis in adults [46,47]. Indeed, activins and BMPs use overlapping receptors and share downstream signaling with TGF-β [48,49,50]. BMP7, however, plays a controversial role in fibrosis induction [51,52]. CCL2 was found to be upregulated in SSc patients and its levels correlate with the SSc symptom score [53]. CCL3 is similarly increased in the skin of SSc subjects [53,54] and can induce fibrosis in an SSc murine model [55]. SERPINA1 is associated with lung fibrosis [56]. TNFα has a recognized role in the induction of fibrosis [57], and its inhibition has an anti-fibrotic effect, reducing skin fibrosis and ameliorating the modified Rodnan skin thickness score (MRSS) [58,59,60,61]. However, although in vivo studies in animal models also support a pro-fibrotic role, in vitro studies have sometimes reported TNFα as an antifibrotic cytokine, not allowing us to draw a definitive conclusion about its role [62]. These differences may be due to the inflammatory components of fibrosis induced by TNFα in vivo, which are lacking in in vitro studies [62]. Elevated levels of PDGF-A and -B expression have been detected in the skin, lung, and endothelial cells of SSc patients; furthermore, they are key players in liver fibrosis, and the data suggest that crosstalk between the TGF-β and PDGF pathways may regulate SSc chronic fibrosis [63]. Interestingly, all these factors were induced at significantly lower levels by HCMV or HHV-6A in vitro individual infection, supporting the synergistic activity of these viruses. 

Coherent with the hypothesized pro-fibrotic action of HCMV/HHV-6A coinfection, down-regulated factors included anti-fibrotic molecules. DCN, an extracellular matrix (ECM) protein, affects a wide range of biological processes, including cell growth, differentiation, proliferation, adhesion, spread and migration; it regulates inflammation and fibrillogenesis, and has a potent antifibrotic effect, antagonizing fibrosis and potentially being proposed for antifibrosis therapy [64]. IL-13Ra2 has an anti-fibrotic effect, as its overexpression inhibits the expression of fibrotic markers in vitro and in pulmonary fibrosis in vivo [65]. A decrease in the activity of MMPs, including MMP2, in SSc fibroblasts causes extracellular matrix deposition and fibrosis development [66,67]; moreover, MMP2 exhibits direct anti-fibrotic activity in murine models [68]. TIMP-3 was variably reported to be increased or decreased in SSc studies; thus, its association with fibrosis is controversial [69]. THBS-2 expression was down-regulated in vitro in SSc dermal fibroblasts; on the other hand, it was found to be increased in the skin and serum of subjects with scars and/or ulcers [70]. Its silencing downregulates type I collagen synthesis in SSc fibroblasts, and recent data suggest that the extracellular presence of THBS-2 could promote a pro-fibrotic environment in SSc [70]. HGF also plays a controversial role in fibrosis, since some studies show its inhibitory effect on lung fibrosis by antagonizing TGFβ [71] but other studies report HGF up-regulation in SSc skin [72] and increased HGF production in SSc fibroblasts compared with normal fibroblasts [73]. 

Finally, a few factors down-regulated by coinfection were instead reported to have a clear pro-fibrotic effect, so the meaning of their down-regulation by coinfection is unclear. They include FASLG, COL1A1, COL3A1, and LOX, whose down-regulations were, however, observed only at late time points, perhaps as a result of the overstimulation of other factors. Among them, FASLG plays a key role in pulmonary fibrosis by inducing inflammatory apoptosis in epithelial cells and alveolar macrophages, and its blockade can prevent or attenuate lung fibrosis [74]. COL1A1 and COL3A1 are up-regulated in SSc dermal fibroblasts, and their silencing has been observed to attenuate fibrosis in a SSc mouse model [75,76]. LOX has been found to be increased in lung fibroblasts of SSc patients and in a murine model of pulmonary fibrosis, suggesting its direct pathogenic role in SSc-associated fibrosis [77]. 

Among apoptosis-related factors, HCMV/HHV-6A coinfection induced significant up-regulation of 12 factors: BCL2L10 (+4.76-fold), BID (+7.02-fold), BIK (+113.46-fold), BIRC3 (+4.39-fold), BIRC5 (+23.72-fold), CD40 (+4.38-fold), HRK (+3.95-fold), TNF (+23.35-fold), TNFRSF1B (+6.03-fold), TNFRSF9 (+8.78-fold), TNFRSF10A (+7.82-fold), and CD27 (+6.25-fold). Conversely, 13 factors were down-regulated moderately: ABL1 (-2.67-fold), BCL2 (-2.04-fold), BCL2L1 (-2.64-fold), BNIP3L (-2.74-fold), FAS (-3.35-fold), MCL1 (-2.84-fold), NFKB1 (-2.89-fold), NOL3 (-2.52-fold), PYCARD -7.56-fold), RPLP0 (-3.21-fold), TNFRSF11B (-2.69-fold), TNFRSF21 (-3.19-fold), and XIAP (-2.67-fold). In addition, some factors showed alternate up- and down-regulation, including BCL2A1, CD27, TNFSF10, and CASP1. 

Almost all up-regulated factors have documented pro-apoptotic activity: BCL2L10 has a pro-apoptotic effect and its silencing promotes cell growth [78]; BID is known to induce caspase activation and apoptosis [79]; BIK, which was uniquely and potently (>100-fold) up-regulated in coinfected cells and not in individually infected cells, is the most important member of the BH3-only family of pro-apoptotic proteins, and is predominantly localized in the ER, inducing apoptosis and non-apoptotic cell death, mobilizing calcium ions and activating the mitochondrial apoptotic pathway [80]; CD40, a TNF receptor family member, induces cell apoptosis through the activation of caspases 8 and 3, and CD40 ligation was found to induce functional FASL and TNF [81]; HRK (Harakiri, BCL2-interacting protein) encodes a member of the BCL-2 protein family localized to intracellular membranes and promotes apoptosis by interacting with the apoptotic inhibitors BCL-2 and BCL-X(L) via its BH3 domain [82]; and TNF, upregulated >20-fold by coinfection, is one of the best known and most potent inducers of cell apoptosis in the TNF superfamily [83], through interaction with TNFRSF9 and TNFRSF10A [84,85]. By contrast, BIRC3 and BIRC5, somehow stimulated by coinfection, favor evasion from apoptosis [86]. 

Downregulated factors were generally poorly and not constantly decreased by coinfection (<3-fold), so the meaning of their slightly decreased expression in coinfected cells should be interpreted with care. The group of downregulated factors, however, included both pro- (ABL1, BNI3L, FAS, PYCARD, RPLP0, and TNFRSF21) and anti-apoptotic products (BCL2, BCL2L1, MCL1, NFKB1, NOL3, TNFRSF11B, and XIAP), whose final balance, in the presence of a high expression of upregulated pro-apoptotic factors, may more likely push the coinfected cell toward apoptotic death.

Interestingly, recent data reported that SSc fibroblasts are resistant to apoptosis [87] and that survivin (also known as BIRC5, a member of the IAP family, together with BIRC3 and XIAP) could play an important role in apoptosis resistance through caspase inhibition [88,89]. In particular, IAP factors have been reported to confer resistance to the apoptosis induced by TGF-β, which is the major molecular hallmark of SSc, strongly associated with fibrosis [90]. XIAP was also involved in the blockade of TGF-β pro-apoptotic signaling [91] and survivin showed a cell-type-dependent effect [92]. Thus, despite the fact that virus infection can induce apoptosis in fibroblast cells, this might be less important with respect to the induction of fibrosis, due to the observed resistance of SSc fibroblasts to apoptosis [87].

**Figure 4:**
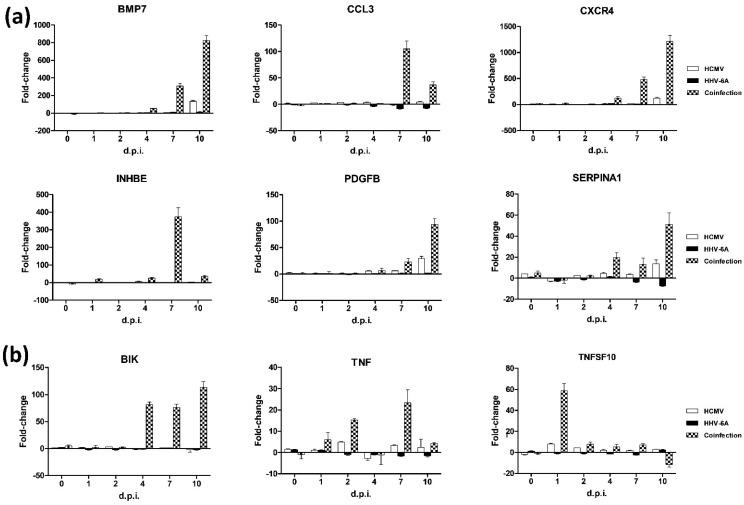
Factors uniquely or mostly affected by viruses’ coinfection. (**a**) Fibrosis-associated factors; (**b**) apoptosis-associated factors. Results are expressed as mean values ± S.D. of duplicate samples in two independent experiments.

Of note, some quantitative differences were noticed between the expression level of factors in the individually infected cells of the present work with respect to what was previously reported by us [13,15], likely due to interassay variability in the human primary dermal fibroblasts used. However, the trend and type of factors altered by HCMV or HHV-6A infection were confirmed, further highlighting the potential role of such viruses and the remarkably enhanced impact of coinfection compared with the individual viruses on the alterations possibly leading to cell fibrosis. Moreover, the activation or deactivation of several factors was suggestive of a true synergism between the viruses, rather than a mere additive effect, as several fibrosis- and apoptosis-associated factors were prominently affected only by coinfection and not (or to a substantially lesser extent) by individual viruses (Figure 4).

The limitations of this study include the use of the microarray technique, which can identify around 80 factors but does not allow for a comprehensive analysis of the expression of intracellular factors related to fibrosis and apoptosis pathway, and the lack of individual qPCR validation of each factor whose expression was altered by virus coinfection. However, the results obtained in single-infected cells confirmed what was previously detected [13], supporting the reliability and reproducibility of the observed effects. Moreover, more mechanistic studies would be needed to understand what viral genes or proteins are responsible for the observed alterations and clarify the pathways involved.

## 5. Conclusions

SSc disease still has an unclear etiology and few therapeutic options. Among the possible environmental triggers of the disease in the susceptible host, β-herpesvirus infection has often been hypothesized based on data reporting the effects of the individual viruses. Herein, for the first time, the collected results show that HCMV and HHV-6A may cooperate in inducing alterations of the expression of fibrosis- and apoptosis- associated factors. Although direct validation of each altered factor will be needed, this is also suggestive of a true cooperation between the two ubiquitous viruses in vivo, thus further supporting their joint role in SSc. Of course, further work is required, both in vitro and ex vivo, to clarify the mechanisms by which changes arise inside infected cells and to definitively answer whether β-herpesviruses can be considered causal agents of SSc, thus pointing to the use of antiviral treatments to improve SSc clinical outcomes.

## Figures and Tables

**Table 1:** HCMV and HHV-6A genome DNA presence in single-infected and coinfected dermal fibroblasts at the indicated days post infection (d.p.i.).

Single Infections			Coinfections	
d.p.i.	HCMV (Copies/µg) ^1^	HHV-6A (Copies/µg) ^1^	HCMV (Copies/µg) ^1^	HHV-6A (Copies/µg) ^1^
0	-	-	-	-
1	3.976 ± 0.002	5.012 ± 0.002	5.017 ± 0.005	5.560 ± 0.004
2	4.767 ± 0.003	5.340 ± 0.004	5.532 ± 0.004	6.227 ± 0.006
4	5.021 ± 0.001	5.745 ± 0.003	7.092 ± 0.003	7.307 ± 0.007
7	5.468 ± 0.005	4.993 ± 0.001	7.977 ± 0.002	5.748 ± 0.001
10	5.651 ± 0.004	4.750 ± 0.002	8.690 ± 0.004	5.470 ± 0.003

^1^ Results are expressed as Log_10_ of mean values of genome copies/µg DNA ± SD.

## Data Availability

All the data supporting the reported results are included in the present paper and in the supplementary materials.

## Supplementary Materials

The following supporting information can be downloaded at: https://www.mdpi.com/article/10.3390/microorganisms10081600/s1, Table S1: Expression of fibrosis-associated factors in single-infected and double-infected cells; Table S2: Expression of apoptosis-associated factors in single-infected and double-infected cells.
