# Supplementary material for: Human Cytomegalovirus and Human Herpesvirus 6 Coinfection of Dermal Fibroblasts Enhances the Pro-Inflammatory Pathway Predisposing to Fibrosis: The Possible Impact on Systemic Sclerosis
Source: Microorganisms. 2022 Aug 8;10(8):1600. doi: 10.3390/microorganisms10081600 (PMC9415275; doi:10.3390/microorganisms10081600)
Supplement: Supplementary file 1 [file microorganisms-10-01600-s001.zip › Table S1 REV.pdf]

Table S1. Fold-change values of fibrosis-associated factors after single or double infection with HCMV and HHV-6A.

Factor	Fold-change expression of analyzed factors (*)																	
	0 d.p.i.			1 d.p.i.			2 d.p.i.			4 d.p.i.			7 d.p.i.				10 d.p.i.	
	HCMV	HHV-6A	HCMV/ HHV-6A	HCMV	HHV-6A	HCMV/ HHV-6A	HCMV	HHV-6A	HCMV/ HHV-6A	HCMV	HHV-6A	HCMV/ HHV-6A	HCMV	HHV-6A	HCMV/ HHV-6A	HCMV	HHV-6A	HCMV/ HHV-6A
ACTA2	1.55	-1.12	-1.02	-1.07	-1.05	1.11	-1.24	-1.12	-1.35	1.64	**-2.07**	-1.5	**2.48**	-1.64	**-5.11**	**2.19**	1.02	**-8.6**
AGT	1.25	**-2.94**	-1.53	1.02	1.37	**4.15**	1.09	-1.04	1.29	**-2.14**	**2.33**	**2.16**	**-2.1**	-1.74	1.65	**2.15**	1.15	**2.41**
AKT1	-1.19	-1.12	1	1.21	1.18	1.47	1.18	-1.01	1.11	-1.8	1.17	1.11	1.09	-1.34	1.21	**2.22**	-1.14	1.12
BCL2	1.42	-1.08	-1.2	1.11	1.25	1.89	-1.31	-1.36	-1.37	**-2.34**	1.2	1.15	1.26	1.11	**-12.28**	-1.23	1.01	-1.36
BMP7	**2.44**	**2.3**	**-11.94**	**2.12**	**2.25**	**3.61**	**2.36**	**2.94**	**3.85**	**3.65**	**4.19**	**53.91**	**5.07**	**9.38**	**308.39**	**134.1**	**13.68**	**823.2**
CAV1	-1.08	1.06	-1.01	-1.19	-1.01	-1.53	-1.12	1.05	-1.1	1.24	-1.58	-1.68	-1.05	1.37	**-4.64**	**-3.05**	-1.36	**-7.12**
CCL11	**2.9**	-1	-1.14	1.81	-1.23	**3.9**	1.82	**2.11**	**2.36**	**3.1**	**-2.6**	1.2	1.66	**-2.13**	-1.63	**2.92**	-1.2	**-4.12**
CCL2	**2.08**	1.27	1.55	**2.13**	-1.5	**9.94**	1.3	-1.22	1.2	**3.13**	1.09	**14.08**	**3.1**	1.86	**8.02**	**2.29**	**2.42**	**-5.47**
CCL3	1.18	-1.08	**-2.56**	**2.46**	1.04	1.32	**3.42**	-1.13	1.97	**3.96**	**-3.7**	1.19	-1.05	**-8.28**	**104.94**	**4.74**	**-6.96**	**36.71**
CCR2	1.06	1.71	**-3.78**	**2.09**	**2.02**	1.79	**2.96**	**2.64**	**3.46**	**2.4**	1.26	1.05	-1.09	**-2.25**	**-6.27**	1.67	1.31	**-4.08**
CEBPB	1.09	1.22	1.4	-1.27	1.1	1.86	-1.15	1.54	1.02	-1.25	1.07	1.04	**2.85**	-1.72	-1.03	**2.56**	-1.18	1.1
COL1A2	1.47	-1.38	1.26	-1.63	1.48	1.02	-1.96	1.16	-1.63	1.2	-1.21	-1.38	1.21	-1.26	**-16.67**	**-3.45**	1.01	**-47.25**
COL3A1	-1.28	-1.24	-1.06	-1.86	1.12	-1.26	-1.51	1.08	-1.47	1.99	1.12	-1.64	**2.22**	1.5	**-29.4**	**-3.7**	1.53	**-113.98**
CCN2	-1.11	-1.2	1.22	1.22	1.03	1.05	1.19	-1.3	-1.22	**-2.42**	-1	-1.05	**2.36**	1.07	1.12	**2.34**	1.62	-1.07
CXCR4	-1.59	**11.12**	**19.13**	**11.4**	1.04	**23.85**	**6.58**	1.6	**10.1**	**15.82**	**21.04**	**127.75**	**18.89**	**11.29**	**482.93**	**123.08**	**6.19**	**1217**
DCN	-1.12	-1.2	1.02	-1.64	1.33	1.62	-1.75	1.13	-1.91	**-2.51**	**2.01**	-1.85	**-2.18**	**2.04**	**-4.3**	**-10.9**	**2.77**	**-78.5**
EDN1	1.37	1.29	1.53	-1.08	-1.02	1.25	-1.14	-1.06	-1.35	**-2.33**	**-2.04**	**-2.21**	-1.34	-1.78	**-2.58**	-1.91	-1.41	-1.31
EGF	1.02	**-2.09**	**-7.59**	1.63	1.36	**3.04**	-1.04	-1.89	1.07	**2.12**	-1.71	1.73	1.06	1.06	-1.81	1.4	1.15	**-2.02**
ENG	1.18	-1.27	1.07	-1.01	1.2	1.47	-1.09	1.09	-1.06	**2.12**	1.02	1.39	**2.31**	-1.17	1.55	**2.2**	-1.17	-1.04
FASLG	1.15	1.14	**-9.49**	**2.46**	**2.05**	**3.2**	-1.4	-1.18	-1.2	**2.48**	1.18	**2.27**	1.58	1.38	**-2.04**	**2.77**	1.65	**-11.61**
GREM1	-1.07	-1.07	1.06	-1.03	1.32	**2.2**	-1.22	1.05	-1.22	**2.24**	1.03	1.13	**2.89**	-1.18	1.18	-1.2	-1.24	**-3.6**
HGF	-1.11	-1.08	-1.09	-1.41	1.4	1.15	-1.53	-1.09	-1.56	**3.59**	1.57	**-3.66**	**4.34**	**2.92**	**-16.54**	-1.6	**2.99**	**-610.49**
IFNG	**-4.99**	-1.95	**-7.96**	**2.8**	**2.95**	1.74	-1.62	-1.95	1.19	**2.27**	1.18	1.58	1.32	1.38	1.05	**2.38**	1.65	**-3.32**
IL10	-1	1.14	1.04	1.35	**-2.33**	1.59	-1.44	-1.81	-1.41	-1.15	1.18	-1.28	**2.55**	**2.62**	-1.49	**13.64**	1.65	-1.07
IL13	1.33	-1.39	**-9.55**	1.01	1.16	1.51	-1.35	-1.43	1.06	**2.39**	-1.92	1.01	**2.22**	**-2.33**	**-2.35**	**9.61**	1.09	-1.4
IL13RA2	-1.14	-1.1	-1.13	-1.19	1.02	-1.42	-1.49	-1.05	-1.43	**-3.42**	-1.03	-1.31	**-5.57**	1.89	**-6.16**	**-8.44**	1.6	**-31.48**
IL1A	1.96	1.35	-1.51	1.3	1.4	-1.46	1.32	-1.63	**-2.1**	1.51	**-3.12**	**3.63**	1.5	-1.31	1.38	**2.86**	-1.22	1.25
IL1B	**3.36**	-1.07	**-5.12**	**2.69**	**2.93**	**2.96**	**3.68**	-1.69	1.4	**4.08**	**4.56**	1.23	**4.04**	**3.88**	**7.73**	**2.37**	**3.05**	**2.19**

IL4	**2.02**	-1.68	**-3.26**	-1.94	**2.94**	**-2.1**	1.41	-1.15	-1.93	1.81	1.18	1.97	**6.58**	**8.34**	**-7.79**	**3.14**	**7.36**	**-2.41**
IL5	1.66	1.19	**-9.63**	**2.74**	1.22	**2.85**	**3.54**	**-2.59**	**3**	**2.49**	-1.89	**-2.88**	**2.07**	1.65	**-3.75**	-1.43	1.41	**3.53**
ILK	-1.05	1.08	1.02	-1.04	-1.04	-1.15	-1.03	-1.02	1.03	1.03	-1.03	-1.01	1.39	-1.03	1.29	1.95	-1.33	1.62
INHBE	1.02	**-2.4**	**-6.58**	-1.42	1.2	**18.62**	-1.28	-1.28	-1.28	**6.93**	1.18	**26.26**	1.12	**-2.23**	**373.75**	**2.87**	-1.92	**35.52**
ITGA1	-1.18	1.25	1.1	1.36	1.15	**2.05**	1.25	1.03	1.41	**2.03**	1.05	1.32	**2.85**	**2.56**	1.34	**2.11**	**2.39**	1.17
ITGA2	-1.23	-1.16	1.02	**2**	1.08	**3.25**	1.65	1.07	1.61	1.72	1.5	1.12	**2.51**	**2.09**	**2.13**	1.52	1.36	1.46
ITGA3	1.09	1.04	1.14	**2.03**	1.26	**2.54**	1.51	-1.06	1.82	**2.09**	**-2.21**	**2.83**	1.09	**-2.97**	**6.55**	**3.74**	**-3.36**	**8.53**
ITGAV	-1.08	1.01	1.06	-1.2	1.13	1.41	-1.33	1.19	-1.45	**-2.04**	1.42	-1.32	1.15	1.82	**-2.9**	**-2.32**	1.82	**-4.44**
ITGB1	-1.14	-1.02	1.13	-1.09	1.11	1.37	-1.39	1.02	-1.1	-1.05	1.06	-1.42	1.26	**2.08**	**-2.49**	**-2.7**	1.21	**-3.35**
ITGB3	-1.14	-1.12	-1.04	-1.15	1.19	1.12	-1.16	-1.02	1.06	**2.7**	-1.61	1.04	**2.09**	-1.11	-1.82	1.35	-1.26	**-2.39**
ITGB5	-1.22	-1.05	1.1	-1.15	1.11	1.2	-1.28	1.13	-1.16	**-2.1**	1.49	-1.05	**2.6**	-1.54	-1.85	**2.64**	-1.38	**-2.16**
ITGB6	1.11	-1.16	**-5.34**	1.28	1.26	1.51	-1.88	-1.56	-1.46	**2**	**2.52**	1.07	1.37	1.38	-1.4	-1.62	1.67	1.39
ITGB8	-1.13	-1.19	-1.21	1.13	1.07	**2.26**	1.05	-1.11	1.13	**-2.63**	**2.08**	1.31	-1.35	**2.94**	1.51	**-2.68**	**2.29**	-1.83
JUN	-1.05	1.17	1.26	1.08	1.27	1.12	-1.22	1.01	-1.26	1.27	1.6	-1.41	**2.25**	-1.29	**-2**	-1.5	-1.59	**-2.11**
LOX	-1.22	-1.09	1.03	-1.24	-1.02	1.1	-1.07	1.06	-1.02	**-2.28**	-1.23	-1.36	-1.2	1.48	**-3.8**	**-5.3**	1.26	**-28.44**
LTBP1	-1.15	-1.08	-1.11	-1.14	1.03	-1.08	-1.42	1.06	-1.33	**3.16**	-1.21	-1.33	**2.55**	**2.12**	**-2.2**	1.44	1.38	**-5.41**
MMP1	-1.24	1.05	1.01	1.33	1.25	1.69	-1.14	-1.04	1.12	**3.51**	1.09	**2.08**	1.39	**2.51**	**5.65**	**-2.11**	-1.09	-1.46
MMP13	-1.05	-1.89	1.54	**3.6**	1.04	**4.16**	1.11	-1.13	**7.51**	**2.51**	1.18	-1.28	**2.44**	1.38	**4.02**	**2.13**	1.65	1.34
MMP14	-1.44	-1.33	1	1.04	1.36	1.82	-1.39	1.31	1.03	-1.85	**2.03**	1.17	**2.93**	-1.19	-1.56	-1.34	1.29	**-6.43**
MMP2	-1.11	-1.39	1.35	1.04	1.71	**2.71**	-1.52	1.05	-1.18	-1.38	1.52	1.36	1.8	1.01	-1.07	-1.77	1.3	**-11.31**
MMP3	-1.29	1.02	1.16	1.07	-1.04	1.2	-1.01	-1.18	1.06	**2.7**	-1.44	1.18	**2.39**	1.75	1.03	**-2.3**	**-2.74**	**-3.53**
MMP8	-1.09	-1.32	**-5.59**	1.54	1.31	**2.15**	-1.4	-1.49	-1.19	**2.81**	**2.35**	1.44	1.12	**2.12**	**-2.21**	**2.37**	**2.79**	-1.97
MMP9	**3.39**	1.18	**-4.52**	**4.15**	-1.01	1.79	**2.07**	1.28	**-3.57**	**2.1**	1.18	-1.98	-1.07	1.38	**3.07**	**3.43**	1.65	**5.04**
MYC	-1.03	1.18	1.4	1.08	1	1.19	-1.28	1.01	-1.26	1.25	1.12	-1.54	1.06	1.12	**-2.13**	-1.89	1.24	-1.37
NFKB1	1.26	1.24	1.16	1.25	1.16	1.71	1.01	1.13	1.09	-1.31	1.86	1.06	1.1	1.55	-1.11	1.27	1.74	**-2.14**
PDGFA	1.07	1.28	1.28	1.31	1.26	**2.01**	1.08	-1.11	1.08	**2.65**	1.35	1.52	**3.44**	1.33	**3.73**	**4.18**	1.44	**5.68**
PDGFB	**2.33**	1.06	1.04	1.07	1.04	1.32	1.35	-1.13	1.13	**5.3**	1.18	**6.26**	**6.12**	1.38	**22.93**	**29.46**	1.65	**93.8**
PLAT	-1.02	1.14	-1.03	1.44	1.08	1.02	-1.02	1.1	1.36	**2.33**	1.11	1.75	**2.97**	-1.71	1.27	**2.14**	-1.59	1.51
PLAU	1.95	1.12	1.14	1.04	1.01	1.41	-1.17	-1.15	-1.32	1.32	1.39	-1.05	**2.14**	1.73	-1.37	-1.02	1.66	**-4.35**
PLG	1.31	-1.13	-1.15	**2.27**	1.04	**3.84**	-1.62	**-2.03**	-1.58	**2.39**	1.18	-1.28	**2.89**	**2.99**	1.28	1.71	1.65	**8.5**
SERPINA1	**3.99**	1.06	**4.86**	**-2.63**	**-2.85**	-1.61	**2.67**	-1.22	**2.15**	**4.53**	1.58	**19.77**	**3.64**	**-3.71**	**13.27**	**13.67**	**-7.33**	**51.25**
SERPINE1	1.04	1.09	1.2	1.33	-1.19	-1.18	1.01	1.03	-1.01	-1.61	**-2.4**	1.43	**2.61**	-1.73	1.92	**2.29**	**-2.14**	1.02
SERPINH1	-1.01	-1.09	1.07	-1.35	1.07	1.23	-1.45	-1.1	-1.34	1.32	1.17	1.01	-1.7	1.31	**-3.75**	**-2.38**	1.33	**-2.62**

SMAD2	-1.39	1.14	1.01	-1.01	1.07	1.29	1.03	-1.16	-1.15	-1.43	-1.1	-1.18	-1.34	1.34	-1.12	1.2	1.21	-1.07
SMAD3	1.08	-1.08	-1.17	-1.01	1.21	1.06	-1.35	1.01	-1.14	-1.14	-1.77	-1.01	1.8	**-2.27**	-1.75	**2.59**	**-2.23**	**-2.21**
SMAD4	-1.07	-1.3	-1.09	1.11	1.16	1.56	1.1	1.15	1.07	-1.64	1.03	-1.08	1.07	1.09	-1.32	-1.4	-1.02	-1.67
SMAD6	1.11	-1.18	-1.24	-1.38	-1.1	-1.88	-1.04	1.31	1.14	1.17	1.58	-1.69	1.3	1.56	**-2.24**	-1.18	1.6	**-3.23**
SMAD7	1.28	1.4	1.44	-1.13	1.21	1.13	-1.15	-1.01	-1.29	**-2.21**	1.18	1.2	**2.79**	**-2.08**	1.32	**2.07**	-1.62	1.56
SNAI1	-1	1.57	1.87	-1.07	-1.03	1.46	-1.08	-1.12	-1.2	-1.69	1.08	**2.4**	-1.62	1.15	**4.65**	**2.15**	-1.04	**4.35**
SP1	-1.04	-1.06	-1.09	1.72	1.69	**2.48**	1.23	1.14	1.09	-1.13	1.62	1.05	1.84	1.28	-1.03	1.09	1.78	-1.15
STAT1	-1.16	-1.01	1.02	**3.04**	1.52	**7.78**	**2.96**	-1.09	**3.59**	**3.05**	1.46	**4.36**	1.62	1.92	1.71	-1.55	**2.26**	**-2.03**
STAT6	1.21	-1.37	1.02	1.16	1.07	1.72	-1.18	1.01	1.07	1.06	1.21	1.48	1.7	-1.22	-1.52	-1.1	-1.65	**-3.62**
TGFB1	1.06	-1.09	1.15	-1	1.28	1.41	-1.06	-1.12	1.02	-1.48	-1.08	1.46	1.13	**-2.05**	**2.22**	**2.46**	-1.67	**2.64**
TGFB2	1.06	-1.22	-1.07	-1.97	1.26	-1.27	**-2.04**	1.05	-1.97	**2.92**	1.43	**-2.15**	**3.99**	**3.15**	**-3.43**	-1.18	**2.36**	-1.45
TGFB3	1.16	1.27	-1.07	1.89	-1.25	**2.54**	1.32	-1.32	1.45	**2.04**	-1.15	1.51	1.9	-1.04	1.03	1.29	1.14	-1.49
TGFBR1	1.03	-1.11	-1.13	1.07	-1.01	1.07	1.27	-1.14	1.09	**-2.04**	1.74	1.04	1.46	**2.23**	-1.26	-1.31	**2.71**	-1.2
TGFBR2	-1.54	1.14	1.08	-1.01	1.24	1.38	1.03	1.03	-1	1.79	1.59	-1.04	**2.84**	1.58	-1.61	1.05	1.74	**-4.64**
TGIF1	1.48	-1.13	-1.11	-1.16	1.08	1.4	-1.3	-1.05	-1.24	1.92	1.45	-1.08	1.61	1.34	-1.08	-1.27	1.63	-1.06
THBS1	1.45	1	1.08	-1.12	1.02	1.24	-1.3	-1.05	-1.54	1.39	-1.52	-1.53	**2.41**	-1.71	-1.64	**2.09**	-1.96	**-4.89**
THBS2	1.05	1.01	1.1	-1.5	1.51	1.49	-1.97	1.02	-1.9	**-2.94**	1.81	-1.77	1.42	1.2	**-11.43**	**-3.87**	1.37	**-47.91**
TIMP1	1.44	-1.26	1.13	-1.38	1.11	1.07	-1.35	1.11	-1.34	**2.03**	-1.47	-1.1	**2.3**	1.56	1.06	-1.14	1.11	-1.49
TIMP2	1.52	-1.31	1.11	1.06	1.4	1.51	-1.18	1.2	-1.04	**-2.86**	1.59	1.14	**2.35**	-1.08	-1.86	-1.04	1.01	**-5.41**
TIMP3	1.08	-1.17	1.05	-1.39	1.05	-1.24	-1.61	-1.07	-1.97	**-2.67**	1.01	-1.91	**-2.19**	1.41	**-6.69**	**-3.86**	**2.67**	**-16.67**
TIMP4	-1.24	1.06	1.22	-1.07	-1.48	1.19	1.27	-1.12	1.59	**2.98**	-1.75	1.58	1.78	-1.94	**2.1**	1.32	**-2.73**	**4.23**
TNF	**3.37**	**-2.99**	1.2	1.07	**9.1**	**23.21**	-1.11	**-2.46**	1.39	**3.6**	1.18	-1.28	**10.56**	**5.19**	**26.54**	**34.21**	**27.92**	**2.96**
VEGFA	1.48	1.26	1.68	1.03	1.02	**2.07**	1.54	-1.22	1.1	**2.74**	1.03	-1.05	1.04	1.13	1.71	1.04	1.59	**2.62**

(*) Results are expressed as mean values of fold-change observed in single- or double-infected cells. compared to uninfected control cells. All values exceeding +2 or -2 folds are indicated in bold. Orange boxes indicate >3 up-regulated factors. Blue boxes indicate <-3 down-regulated factors.
