# Supplementary material for: Human Cytomegalovirus and Human Herpesvirus 6 Coinfection of Dermal Fibroblasts Enhances the Pro-Inflammatory Pathway Predisposing to Fibrosis: The Possible Impact on Systemic Sclerosis
Source: Microorganisms. 2022 Aug 8;10(8):1600. doi: 10.3390/microorganisms10081600 (PMC9415275; doi:10.3390/microorganisms10081600)
Supplement: Supplementary file 1 [file microorganisms-10-01600-s001.zip › Table S2 REV.pdf]

Table S2. Fold-change values of apoptosis-associated factors after single or double infection with HCMV and HHV-6A.

Factor	Fold-change expression of analyzed factors (*)																	
	0 d.p.i.			1 d.p.i.			2 d.p.i.			4 d.p.i.			7 d.p.i.				10 d.p.i.	
	HCMV	HHV-6A	HCMV/ HHV-A	HCMV	HHV-6A	HCMV/ HHV-6A	HCMV	HHV-6A	HCMV/ HHV-6A	HCMV	HHV-6A	HCMV/ HHV-6A	HCMV	HHV-6A	HCMV/ HHV-6A	HCMV	HHV-6A	HCMV/ HHV-6A
ABL1	1.15	-1.12	1.27	-1.08	1.3	1.62	-1.19	1.21	-1.13	1.13	1.03	-1.05	-1.01	-1.34	-1.9	1.3	1.01	**-2.67**
AIFM1	-1.04	-1.19	-1.06	1.28	1.12	1.43	1.34	-1.05	1.43	**-2.16**	1.29	1.31	**2.03**	-1.35	1.39	**2.48**	-1.09	**2.06**
AKT1	1.02	1.04	1.11	1.26	1.04	1.34	1.07	1.13	1.35	-1.54	-1.07	1.15	1.72	-1.28	1.36	1.02	1.12	1.18
APAF1	1.07	1.47	1.3	1.4	1.16	1.1	1.06	1.09	1.04	**-2.13**	1.46	-1.16	**2.27**	-1.32	1.33	**2.39**	-1.08	1.33
BAD	1.01	1.03	1.05	-1.01	1.02	1.19	1.01	-1.07	-1.02	1.55	-1.15	-1.02	**2.52**	-1.86	-1.07	1.97	-1.24	1.08
BAG1	1.16	-1.2	-1.17	1.62	1.85	**4.43**	1.56	1.31	1.33	1.71	-1.54	1.79	1.97	-1.75	**2.59**	1.99	-1.19	-1.92
BAG3	1.22	1.06	1.31	**2.24**	1.24	1.68	-1.01	-1.02	1.18	1.83	-1.14	1.5	1.98	-1.73	1.96	**2.38**	-1.23	**3.15**
BAK1	1.05	1.09	1.22	1.45	-1.01	1.84	1.72	1.22	1.65	-1.23	1.01	1.27	**2.17**	-1.5	**3.33**	1.1	-1.15	**2.35**
BAX	-1.18	-1.11	1.1	1.23	1.18	1.33	1.02	-1.04	1.26	-1.56	-1.01	1.19	1.37	-1.08	-1.04	-1.4	1.06	-1.8
BCL10	1.03	1	-1.16	1.42	1.06	1.32	1.03	-1.11	-1.01	**-2.82**	1.13	-1.15	1.94	-1.66	1.12	-1.72	-1.05	-1.05
BCL2	1.97	1.26	1.33	1.08	1.15	1.71	-1.49	-1.09	-1.47	**2.29**	**2.31**	-1.41	**3.89**	**5.42**	**-2.04**	1.35	1.18	-1.37
BCL2A1	-1.26	1.77	**4.87**	-1.36	-1.51	**7.18**	-1.43	**-2.71**	**-3.49**	-1.89	-1.1	**14.66**	**2.92**	**-2.73**	**3.99**	1.33	**-2.01**	-1.08
BCL2L1	-1.39	-1.07	1.12	1.12	1.02	1.14	-1.13	1.01	-1.11	-1.22	**-2.35**	-1.2	**2.78**	**-2.1**	-1.16	**2.89**	-1.31	**-2.64**
BCL2L10	1.05	1.44	1	1.16	1.02	1.39	**3.6**	-1.03	**2.1**	1.34	1.87	1.04	**2.95**	-1.13	**4.42**	**2.69**	1.48	**4.76**
BCL2L11	1.52	1.35	1.17	1.54	1.08	**3.35**	**2.59**	1.16	1.51	-1.6	-1.23	-1.62	1.43	**-2.28**	1.08	-1.11	-1.84	-1.05
BCL2L2	-1.02	1.89	1.77	1.51	1.29	**2.16**	1.25	1.06	1.32	-1.06	-1.14	1.12	1.93	-1.61	1.31	1.17	-1.2	-1.11
BFAR	1.1	1.27	1.39	1.08	1.07	1.45	1.06	-1.18	-1.07	1.33	-1.32	-1.07	1.72	-1.76	-1.12	1.92	-1.1	-1.31
BID	1.06	1.12	1.32	1.38	1.05	**2.16**	-1.01	-1.06	1.21	**-2.23**	1.02	**2.93**	**3.26**	**-2.44**	**7.02**	**5.1**	1.7	**6.43**
BIK	1.06	**2.18**	**4.87**	1.74	**-2.38**	**2.25**	**2.86**	**-2.44**	**2.27**	-1.17	-1.31	**81.8**	1.02	-1.03	**76.28**	**-2.49**	1.35	**113.46**
BIRC2	1.01	1.08	1.37	1.14	1.01	1.77	-1.17	-1.05	-1.02	**-2.13**	1.9	-1.19	1.38	**2.24**	-1.27	**-2.46**	**2.97**	-1.9
BIRC3	1.43	1.19	1.82	1.48	1.02	**3.57**	-1.87	-1.11	-1.59	**-3.05**	1.83	1.95	**-3.45**	**-5.78**	**4.39**	**2.64**	**2.82**	-1.28
BIRC5	-1.3	-1.38	-1.2	1.35	1.15	**-2.26**	**2.35**	-1.02	**2.4**	-1.63	-1.02	**23.72**	1.17	1.8	**2.83**	-1.95	**2.48**	**3.01**
BIRC6	-1.21	1.01	1.09	1.22	1.04	1.15	1.05	-1.06	-1.18	-1.98	1.11	-1.3	-1.1	1.31	1.1	**-2.8**	1.99	1.11
BNIP2	-1.38	1.13	1.1	1.17	-1.05	1.38	1.15	-1.06	1	-1.16	-1.07	-1.04	1.21	-1.16	-1.17	-1.24	1.19	-1.24
BNIP3	-1.17	1.09	1.09	1.06	1.02	1.26	-1.09	-1.26	-1.04	-1.85	1.33	-1.07	1.31	1.11	1.06	**-2.71**	1.61	1.02
BNIP3L	-1.14	1.16	1.1	1.01	1.05	1.44	-1.13	-1.03	-1.32	**-2.15**	-1.03	-1.48	-1.41	1.15	**-2.51**	-1.32	1.29	**-2.74**
BRAF	-1.33	1.11	1.15	-1.15	-1.03	1.28	-1.07	1.02	1.44	**2.13**	1.04	1.54	1.26	1.07	1.87	**-2.52**	1.6	1.05
CASP1	1.27	-1	1.01	**3.63**	1.27	**7.88**	**2.35**	-1.11	**2.46**	1.41	1.13	**2.74**	**2.83**	1.06	1.24	**-4.38**	1.17	**-49.92**

CASP10	1.06	-1.25	-1.01	1.41	1.1	**2.45**	1.53	-1.16	1.46	1.22	-1.17	1.39	1.67	-1.28	1.01	**2.93**	1.18	**-2.16**
CASP14	1.11	-1.03	**2.04**	1.91	1.82	**3.95**	1.64	-1.01	1.23	-1.24	1.03	**2.84**	**2.25**	-1.54	-1.16	**2.17**	-1.28	-1.11
CASP2	-1.24	-1.02	1.07	1.83	1.14	1.61	1.68	-1.14	1.64	-1.3	1.15	1.22	1.88	-1.33	1.82	-1.26	1.28	1.81
CASP3	-1.2	1.01	1.15	1.18	-1.12	1.52	1.35	1.21	1.39	1.78	-1.09	-1.06	**2.05**	1.1	**2.71**	**-2.31**	1.61	-1.14
CASP4	**-2.83**	1.02	1.11	1.53	1.03	**2.2**	1.15	-1.16	1.17	**2.09**	**2.44**	1.28	1.53	**2.05**	1.18	**-2.59**	1.18	-1.55
CASP5	-1.01	-1.91	**-4.81**	-1.42	**-2.55**	**-3.67**	1.07	-1.02	1.14	1.03	1.16	-1.15	1.63	-1.43	**2.97**	**-2.11**	1.05	1.37
CASP6	1.18	1.12	1.03	1.4	1.02	1.37	1.49	1.1	1.39	-1.55	1.37	-1	**2.45**	-1.1	-1.06	**-2.02**	1.1	-1.47
CASP7	-1.58	1.01	1.07	**2.23**	1.47	**2.86**	1.29	-1.08	1.22	**2.28**	-1.1	1.47	**2.01**	-1.1	1.07	-1.74	1.29	**-2.13**
CASP8	1.49	-1.06	1.01	1.58	1.01	**2.11**	1.31	-1.05	1.79	1.6	-1.49	1.04	1.47	**-2.42**	-1.14	-1.2	-1.49	**-2.46**
CASP9	**-2.62**	-1.05	1.05	-1.03	1.01	1.26	1.02	-1.04	1.19	-1.11	**2.25**	1.18	**2.1**	**-2.53**	-1.24	-1.12	**5.13**	-1.24
CD27	1.31	-1.21	-1.75	**3.68**	**3.47**	**3.84**	1.58	**2.29**	**5.09**	**3.58**	-1.07	-1.6	**14.42**	**-2.56**	**6.25**	**7.06**	**-2.53**	**-4.05**
CD40	1.06	-1.5	-1.21	1.38	1.2	**3**	1.05	-1.02	1.22	-1.22	-1.17	1.71	-1.38	-1.28	**4.38**	**2.22**	**2.09**	**2.55**
CD40LG	1.87	1.23	**2.06**	1.31	1.6	**2.1**	1.1	-1.17	1.13	1.39	-1.14	1.24	1.93	**-2.52**	**-2.01**	1.85	-1.23	1.59
CD70	1	-1.18	1.5	1.2	1.74	**2.72**	1.41	1.34	1.21	1.78	1.12	1.69	**2.37**	-1.04	1.71	**-2.04**	1.52	1.1
CFLAR	1.12	1.15	1.05	1.46	1.19	**2.4**	1.03	-1.05	1.01	1.22	**2.81**	-1.19	1.46	**-2.51**	-1.28	1.52	**-2.95**	**-3.85**
CIDEA	-1.02	**-2.82**	1.25	-1.02	**-2.71**	-1.29	1.45	**-2.69**	1.36	-1.04	-1.16	**2.68**	**2.4**	-1.83	-1.94	**2.08**	-1.26	1.08
CIDEB	1	-1.06	1.1	1.45	1.11	1.53	1.09	-1.27	1.08	-1.22	-1.2	1.16	**2.15**	-1.79	1.64	1.12	-1.43	**2.66**
CRADD	-1.42	1.13	1.1	-1.03	-1.17	-1.1	1.37	-1.01	1.05	-1.25	1.19	-1.29	**2.1**	1.51	1.06	1.72	1.51	**2.19**
CYCS	1.17	-1.92	-1.32	**2.1**	1.16	1.72	1.03	-1.8	1.11	**2.01**	1.03	1.16	**3.61**	-1.99	-1.76	**2.84**	1.35	-1.14
DAPK1	1.01	1.58	1.15	1.02	1.42	**2.18**	-1.11	-1.08	-1.37	1.43	-1.33	-1.09	**2.72**	-1.78	1.07	1.19	-1.47	**2.25**
DFFA	-1.08	1.17	1.12	1.35	1.08	1.36	1.1	-1.21	1.07	**2.07**	1.26	1.21	**2.87**	**-2.13**	1.54	**2.02**	-1.51	1.78
DIABLO	-1.01	-1	-1.17	-1.21	-1.2	1.04	1.07	-1.03	1.12	**2.88**	1.29	-1.1	**2.11**	**-2.03**	1.27	**2.64**	-1.4	1.61
FADD	1.25	1.09	1.15	1.34	1.11	1.53	1.29	-1.03	1.29	1.82	1.24	1.24	1.57	1.43	1.9	**-2.89**	**2.36**	1.35
FAS	1.06	1.15	1.13	1.34	1.02	1.78	1.12	1.03	1.09	1.22	1.01	1.05	1.67	-1.28	-1.12	**2.93**	**3.7**	**-3.35**
FASLG	1.19	-1.87	-1.26	1.33	1.67	**2.33**	1.76	-1.32	**2.32**	-1.74	1.74	1.21	**2.92**	-1.3	**-2.15**	-1.84	1.15	1.82
GADD45A	-1.1	-1.24	-1.13	1.05	1.05	1.36	1.26	-1.03	1.18	1.33	1.07	-1.16	-1.23	**-2.34**	-1.34	1.33	-1.21	1.01
HRK	-1.12	1.09	**-2.64**	-1.73	1.36	**3.95**	1.1	-1.01	-1.16	**2.08**	1.3	**2.05**	**2.38**	-1.42	**2.52**	-1.22	-1.05	**2.85**
IGF1R	1.06	1.03	1.06	-1.02	1.07	**2.27**	-1.19	-1.43	-1.35	1.22	-1.17	-1.3	1.67	-1.28	**-2.18**	**-3.65**	1.18	**-2.05**
IL10	**2.41**	-1.7	**-3.63**	**2.32**	-1.16	**2.51**	-1.18	**-2.16**	-1.11	1.22	-1.17	**2.58**	**2.88**	1.4	-1.77	**2.21**	1.18	1.16
LTA	1.06	-1.03	-1.65	1.54	**2.48**	**2.14**	1.06	1.21	1.8	-1.22	1.03	-1.02	**2.05**	-1.19	**-2.23**	1.82	1.04	-1.48
LTBR	1.02	1.18	1.23	1.31	1.53	**2.49**	-1.36	-1.09	-1.12	1.44	-1.22	1.33	1.48	-1.05	1.43	**-2.59**	1.49	-1.04
MCL1	1.19	-1.08	1.07	1.02	1.07	1.63	-1.06	-1.12	-1.03	**3.37**	-1.2	-1.17	**2.35**	-1.05	-1.57	1.39	1.42	**-2.84**
NAIP	1.59	1.59	-1.56	-1.28	1.3	-1.09	1.07	-1.49	1.18	-1.36	1.32	-1.56	**2.44**	-1.26	-1.97	-1.18	1.05	1.03

NFKB1	-1.14	1.21	1.41	1.33	1.08	1.78	-1.02	1.05	1.02	**2.21**	1.42	-1.22	**2.96**	-1.28	-1.34	1.64	1.06	**-2.89**
NOD1	1.01	-1.22	-1.2	**2.39**	1.1	**3.57**	1.66	-1.14	1.5	1.73	-1.12	**2.09**	**2.39**	**-2.42**	**2.63**	1.87	-1.56	1.73
NOL3	1.05	1.09	1.27	1.43	1.22	1.66	1.08	-1.09	1.03	-1.31	-1.89	1.24	-1.14	**-2.83**	-1.75	**-2.39**	**-2.41**	**-2.52**
PYCARD	1.18	1.07	1.08	1.35	-1.02	**2.17**	-1.05	-1.02	1.09	1.16	-1.02	-1.38	1.02	-1.09	**-3.62**	-1.41	1.13	**-7.53**
RIPK2	**2.88**	1.12	1.15	1.25	1.17	**2.31**	-1.06	-1.19	1.1	**6.79**	-1.17	1.13	**4.64**	**3.91**	**2.08**	**2.53**	1.18	1.87
TNF	1.48	1.28	-1.02	1.11	1.07	**5.97**	**4.96**	-1.02	**15.21**	**-2.83**	-1.02	-1.15	**3.33**	-1.55	**23.35**	**2.36**	-1.54	**4.27**
TNFRSF10A	1.43	1.06	1.11	**2.11**	1.16	**3.43**	1.94	-1.18	1.39	-1.17	1.47	**2.38**	**3.24**	-1.55	**7.82**	-1.28	1.34	**5.06**
TNFRSF10B	1.24	-1.03	-1.04	1.21	-1.04	1.71	1.13	-1.09	1.11	-1.82	-1.44	-1.28	1.24	1.31	1	**-2.47**	1.96	-1.35
TNFRSF11B	1.31	-1.16	1.03	-1.29	-1.09	1.06	-1.36	-1.06	-1.47	1.11	1.27	-1.05	**2.25**	-1.8	-1.19	**2.21**	-1.38	**-2.69**
TNFRSF1A	1.38	1.17	1.37	-1.16	1.14	1.27	-1.09	1.04	-1.02	-1.05	-1.02	1.02	**2.21**	**-2.6**	-1.29	**2.27**	-1.17	-1.91
TNFRSF1B	1.05	-1.06	-1.06	1.11	**-2.7**	1.23	1.18	-1.05	1.01	1.58	1.36	**2.05**	1.58	-1.2	**6.03**	**-2.71**	1.23	**3.36**
TNFRSF21	**2.3**	-1.05	1.04	-1.03	1.12	1.65	-1.08	1.04	1.07	1.75	-1.12	-1.16	**2.14**	**-2.39**	**-3.17**	1.97	-1.56	**-2.59**
TNFRSF25	**2.02**	**2.84**	**2.34**	1.11	**-7.21**	-1.18	-1.01	-1.65	1.15	**2.9**	**-14.34**	**-4.58**	**16.48**	**-15.68**	1.43	**14.29**	**-10.37**	**-2.03**
TNFRSF9	1.71	-1.5	-1.25	1.23	**2.5**	**8.66**	1.2	-1.46	**5.7**	**5.17**	**2.03**	-1.06	**4.73**	**2.34**	**8.78**	**-2.43**	**2.19**	**5.57**
TNFSF10	**-2.15**	1.25	-1.12	**8.19**	-1.05	**58.8**	**4.22**	-1.04	**7.77**	**2.24**	-1.51	**5.13**	1.67	**-2.39**	**6.94**	**2.93**	**2.25**	**-11.46**
TNFSF8	1.13	1.37	-1.49	-1.9	**-3.43**	1.91	1.42	**-2.91**	-1.12	-1.3	1.16	-1.08	**2.03**	-1.75	-1.63	1.51	-1.03	**2.68**
TP53	1.1	-1.15	1.09	1.31	1.14	1.54	1.12	-1.11	1.01	-1.4	1.19	1.13	1.64	-1.14	-1.25	1.02	1.49	-1.22
TP53BP2	**2.73**	1.34	1.51	1.13	-1.15	1.63	1.08	-1.03	-1.17	1.22	1.85	1.07	**-2.05**	1.55	**2.07**	**2.11**	1.34	**2.26**
TP73	1.43	-1.09	**-2.14**	1.17	1.38	**2.07**	1.24	-1.14	1.3	**-2.04**	**2.13**	1.32	**2.17**	-1.58	-1.13	**2.59**	-1.4	**4.86**
TRADD	1.29	-1.66	-1.22	1.17	-1.35	1.76	1.08	1.19	1.02	1.14	1.22	1.18	**2.37**	-1.9	1.45	**2.23**	-1.08	1.54
TRAF2	1.42	1.04	1.15	1.55	1.09	1.46	1.21	1.11	1.23	-1.45	1.12	1.74	**2.16**	-1.39	**2.49**	1.75	1.14	**2.34**
TRAF3	-1.19	1.01	1.09	1.29	1.2	1.48	1.11	1.08	1.3	-1.28	-1.13	1.02	1.9	-1.28	1.73	**-2.13**	1.05	1.09
XIAP	1.23	1	1.1	1.07	-1.02	1.6	1.26	-1.02	1.07	-1.85	**-2.14**	1.22	**2.28**	**-2.26**	-1.8	1.48	**-2.43**	**-2.67**

(*) Results are expressed as mean values of fold-change observed in single- or double-infected cells. compared to uninfected control cells. All values exceeding +2 or -2 folds are indicated in bold. Orange boxes indicate >3 up-regulated factors. Blue boxes indicate <-3 down-regulated factors.
